# Microalgae Derived Astaxanthin: Research and Consumer Trends and Industrial Use as Food

**DOI:** 10.3390/foods10102303

**Published:** 2021-09-28

**Authors:** Silvia Villaró, Martina Ciardi, Ainoa Morillas-España, Ana Sánchez-Zurano, Gabriel Acién-Fernández, Tomas Lafarga

**Affiliations:** 1 Department of Chemical Engineering, University of Almería, 04120 Almería, Almería, Spain; svc547@inlumine.ual.es (S.V.); martina.ciardi@studio.unibo.it (M.C.); ame778@ual.es (A.M.-E.); asz563@ual.es (A.S.-Z.); facien@ual.es (G.A.-F.); 2 CIESOL Solar Energy Research Centre, Joint Centre University of Almería-CIEMAT, 04120 Almería, Almería, Spain

**Keywords:** *Haematococcus pluvialis*, *Chlorella zofingiensis*, carotenoids, xanthophylls, pigments, bibliometric analysis, functional foods, bioactive compounds, antioxidant capacity, biotechnology

## Abstract

Astaxanthin is a high-value carotenoid currently being produced by chemical synthesis and by extraction from the biomass of the microalga *Haematococcus pluvialis*. Other microalgae, such as *Chlorella zofingiensis*, have the potential for being used as sources of astaxanthin. The differences between the synthetic and the microalgae derived astaxanthin are notorious: not only their production and price but also their uses and bioactivity. Microalgae derived astaxanthin is being used as a pigment in food and feed or aquafeed production and also in cosmetic and pharmaceutical products. Several health-promoting properties have been attributed to astaxanthin, and these were summarized in the current review paper. Most of these properties are attributed to the high antioxidant capacity of this molecule, much higher than that of other known natural compounds. The aim of this review is to consider the main challenges and opportunities of microalgae derived products, such as astaxanthin as food. Moreover, the current study includes a bibliometric analysis that summarizes the current research trends related to astaxanthin. Moreover, the potential utilization of microalgae other than *H. pluvialis* as sources of astaxanthin as well as the health-promoting properties of this valuable compound will be discussed.

## 1. Introduction

Consumer demands for sophisticated and innovative foods have increased. Moreover, consumers are now more aware of the water–energy–food nexus and the relationship between food and health. In this context, the food industry is making significant efforts to increase the sustainability of food production and to produce foods that are not only tasty but also nutritious, functional, and sustainable.

Microalgae are gaining importance in the food industry, and the number of foods containing microalgae that are launched into the market is increasing every year [1]. These microorganisms have several advantages in terms of the consumption of resources and energy when compared to other foods, and they are rich in bioactive compounds, including high value pigments and polyunsaturated fatty acids [2,3]. In the European Union, the use of microalgae as food must comply with Regulation (EU) 2015/2283 on novel foods. Because of their long history of use, some microalgal strains, such as *Arthrospira* (Spirulina), can be consumed in the EU without the need to comply with this regulation [4]. However, other valuable strains, such as *Schizochytrium* or *Haematococcus*, cannot yet be commercialized as food. However, some bioactive compounds derived from these sources have been approved as human foods. For example, oil that is rich in docosahexaenoic acid (DHA) derived from *Schizochytrium* sp. and astaxanthin derived from *Haematococcus pluvialis* can be consumed up to a certain degree in the EU [5,6]. The latter is the most relevant carotenoid pigment present in aquatic animals, being present in salmon, trout, and lobster, among others [7]. Astaxanthin is important for their pigmentation, and a valuable pigment has essential biological functions, such as protection against the oxidation of polyunsaturated fatty acids (PUFAs), immune response, reproductive behavior, and protection against negative ultraviolet light effects [7]. Because of the many positive health effects of astaxanthin, it was recently suggested by one of the world’s leading market intelligence agencies as one of the three main ingredients to watch and a niche ingredient with strong potential [1]. 

The interest in astaxanthin is supported by a large amount of evidence that suggests that this compound has a strong antioxidant capacity that leads to many positive health outcomes upon consumption [7,8,9,10]. However, the high production cost of natural astaxanthin as well as several technological problems (that are common to all microalga derived products) are currently limiting the mass production of this ingredient. For example, the cost of producing natural astaxanthin using the microalga *H. pluvialis* in the EU is approximately 1500 €·kg^−1^ in Greece and 6400 €·kg^−1^ in the Netherlands [11]. The cost of producing synthetic astaxanthin is around 880 €·kg^−1^; therefore, natural astaxanthin cannot compete in terms of cost (for food applications). However, synthetic astaxanthin cannot be used as food in the EU and in the US, and, therefore, natural astaxanthin produced in the EU and the US is the only permitted ingredient for food and the preferred option for high-end applications [12]. Conversely, in China, a recent study predicted the production cost of natural astaxanthin to be as low as 610 €·kg^−1^, which is encouraging as this value would allow the microalgal process to compete with the synthetic alternative [13]. Most of the microalgal astaxanthin being sold today is produced in Asia, although facilities in the US, Europe, and Israel are also producing this valuable compound. To reduce costs, efforts are being undertaken to increase biomass productivity and astaxanthin accumulation. Genetic engineering is a promising strategy that is being studied by many research groups around the globe [14,15,16]. The optimization of photobioreactors to maximize biomass productivity is also being widely investigated, as well as the utilization of alternative culture media produced using low-value nutrients [17].

The aim of the current review is to highlight the challenges and opportunities of astaxanthin use as a food ingredient and to summarize and discuss the most relevant research and industry findings and achievements on the topic. The health benefits of astaxanthin are also discussed and a bibliometric analysis summarized to identify current research trends related to astaxanthin use in the food industry.

## 2. Microalgae as Food: Current Challenges and Opportunities

Microalgae are gaining increased importance as added-value ingredients in foods. Indeed, several of the most important food companies have developed foods containing microalgal biomass, and the number of products being launched into the market is increasing every year [1]. For example, products containing astaxanthin are already commercially available, most of them sold as food supplements containing 1–8 mg of astaxanthin (Table 1). The most common products that contain microalgae that are being commercialized are snacks, baked goods, and beverages. However, the majority of the microalgal biomass currently commercialized is sold as a food supplement as capsules or as a dried powder. 

These microorganisms have emerged as one of the most promising foods to feed an increasing population. The main reasons include their high photosynthetic efficiency and growth rate, higher than those of terrestrial plants [18], and their very high nutritional value. The composition of microalgal biomass depends on many factors, including environmental conditions, the composition of the culture medium, and operational conditions, among others. The composition of the biomass also depends largely on the produced strain. For example, a hierarchical Bayesian analysis of data compiled from the scientific literature recently concluded that (in general) prokaryotic cyanobacteria have higher protein and carbohydrate and lower lipid content than eukaryotic microalgae. That same study reported that the Cryptophyta have higher protein content than, for example, the Ochrophyta, with a particularly high content of lipids [19]. What makes microalgae especially interesting in terms of human nutrition is not only their protein or lipid content but the quality of the proteins and lipids they produce and also their potential to produce and accumulate bioactive compounds with health-promoting properties. Microalgae have been reported to produce and accumulate polyunsaturated fatty acids, such as eicosapentaenoic acid (EPA) and docosahexaenoic acid (DHA) [20], and carotenoids, such as β-carotene, lutein, zeaxanthin, and astaxanthin [21]. 

Other advantages of microalgae include that they can be produced on non-arable land, and, therefore, they do not compete with conventional crops for land, and that some of them can be produced using seawater. Microalgae are well accepted by consumers as these microorganisms are normally considered as sustainable, nutritious, and safe [22]. However, in general, consumers are not aware of the health and environmental benefits of microalgae consumption and production [22]. For example, a recent study suggested a negative attitude towards microalgal biorefineries in Italy and Spain [23], and, therefore, further efforts are needed to increase consumers’ awareness of the environmental benefits of microalgae.

**Figure 1:**
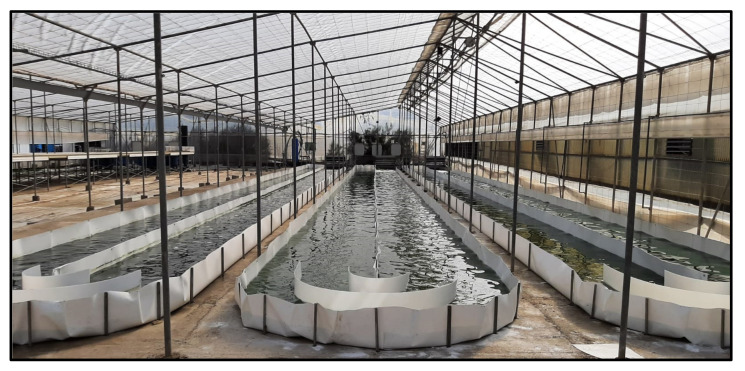
Pilot-scale raceway reactors located inside a greenhouse in Almería, Spain.

The incorporation of microalgae into foods faces some challenges that need to be overcome. The strong “marine” flavor and the intense (generally) green color of microalgae has been suggested as a drawback depending on the traditional diet of the target population [24]. The traditional consumption of algae is common in many countries in Asia but is limited to a few regions in Europe and America. However, the acceptability of foods containing microalgae is generally high [25,26,27,28,29,30,31], and strategies such as masking the color/flavor using ingredients such as spices or chocolate are common [1]. The encapsulation of microalgal biomass was investigated as an efficient method to hide the strong physical characteristics of microalgae [32]. This strategy has the added advantage of promoting the bioactivity and shelf-life of the products. Indeed, the encapsulation of astaxanthin using sodium alginate and pectin [33] or chitosan [34] led to increased shelf-life and improved photoprotection, respectively. One of the main challenges that the industry is currently facing is, although microalgae production is increasing every year, the current capacity is yet several orders of magnitude below the requirements of the food industry. When used as food, microalgae are not consumed alone but as an ingredient introduced into food matrices generally at concentrations ranging from 1–5% *w*/*w* [1]. Still, microalgae production capacity needs to increase to meet the requirements of the food industry. A second important challenge is that microalgae production costs are still high when compared to other food commodities. The cost of producing microalgal biomass depends largely on the location of the reactor and on the overall quality of the biomass. In Europe, the cost of producing microalgal biomass is around 10–50 €·kg^−1^, much higher than that of soya, wheat, rice, or other common food ingredients. For this reason, microalgae are generally produced using raceway reactors, such as those shown in Figure 1, which are cheaper to construct and easier to scale up and operate when compared to closed systems. These are open ponds divided into channels where the culture is recirculated, generally by means of a paddlewheel. The mixing section can include a sump, used to bubble air and carbon dioxide and simultaneously increase mass transfer and remove dissolved oxygen. Their surface-to-volume ratio generally varies between 5 and 10 m^−1^, and their areal biomass productivity is in the range of 20–25 g·m^−2^·day^−1^ when located in areas with sufficient light and adequate temperatures [35].

Closed systems, such as tubular reactors, are easier to control and the quality of the produced biomass is higher, but these are more expensive and, therefore, used for a lower number of applications. These reactors consist of glass/plastic tubes that are used to recirculate the microalgal culture using pumps or air streams. Their surface-to-volume ratio is much higher than that of raceways, generally around 80 m^−1^, allowing much higher biomass productivities to be achieved [36]. As highlighted before, microalgae produce and accumulate valuable compounds, such as polyunsaturated fatty acids, proteins, and carotenoids, rendering the process economically viable. Indeed, the production of high value strains, such as *H. pluvialis*, is conducted using closed systems, as will be discussed below. In this sense, it is important to select not only a productive and robust strain but also one that can provide a benefit to the product.

The current legislation is also delaying the incorporation of microalgae into the food market. As highlighted before, microalgae must be commercialized in the EU under Regulation (EU) 2015/2283, known as the novel foods regulation. Because of their long history of use, Spirulina (*Arthrospira platensis* or *Arthrospira maxima*) and *Chlorella* can be commercialized in the EU without the need to comply with the novel foods regulation. For this reason, most of the products launched into the European market contain biomass of these two strains (mainly Spirulina). The dried biomass of *Tetraselmis chuii* was recently authorized by the European Food Safety Authority (EFSA) as a novel food [37], and products such as *Plancton Marino Veta la Palma* (Fitoplancton Marino S.L., Spain) are currently commercially available. The aim of the novel foods regulation is to ensure that businesses that bring novel and innovative foods to the EU market maintain a high level of food safety for European consumers. However, the commercialization of other strains with potential use as food, such as *H. pluvialis*, *Nannochloropsis*, *Dunaliella*, or *Isochrysis*, among others, is being delayed. Several strains are currently under evaluation, and more will be submitted for assessment in the coming years. This is also happening in the US, where the Food and Drug Administration (FDA) ensures the safety of novel ingredients, and in other countries.

## 3. Astaxanthin, More than Just a Red Pigment

As highlighted before, astaxanthin is mainly used in food and food applications as a pigment because of its intense red color. However, because of the many positive health outcomes attributed to this carotenoid, the research and industrial interest in astaxanthin has increased exponentially.

### 3.1. Astaxanthin Research 2010–2020

**Figure 2:**
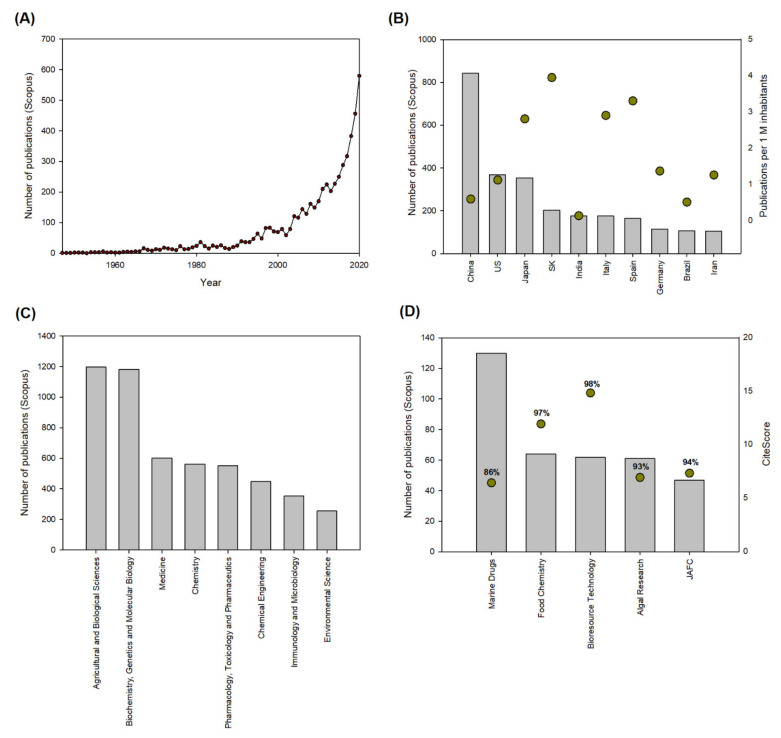
Bibliometric analysis. (**A**) Distribution of publications per year and most relevant (**B**) countries, (**C**) subject areas, and (**D**) source titles of articles published 2010–2020. Abbreviations: US, United States; SK, South Korea; JAFC, Journal of Agricultural and Food Chemistry.

The current section will summarize a bibliometric analysis conducted following a previously described methodology [17]. Briefly, a complete search of the Elsevier Scopus database was carried out using the option “Article title, Abstract, Keywords” and the term “Astaxanthin”. The search resulted in 5715 documents published from 1947 to 2021, as shown in Figure 2A. Overall, the results revealed that the research interest in astaxanthin has increased exponentially during the last few years, with almost 600 publications in 2020 compared to only 69 publications in 2000. These research findings led to the construction of many facilities, and several products containing astaxanthin launched into the market, such as Astapure^®^ (Igennus, Cambridge, UK), which are capsules containing 4 mg of astaxanthin derived from the microalga *H. pluvialis*. These capsules are recommended to be consumed once or twice a day. Recently, the EFSA concluded that an intake of 8 mg of astaxanthin per day from food supplements is safe for adults, even in combination with a high exposure from the background diet [5]. Figure 2B shows the top-10 countries with a higher number of publications including the term astaxanthin either in the title, abstract, or as a keyword. China ranks as the country with the largest number of publications. Indeed, the five institutions with the highest number of publications during the last decade (2010–2020) were: (i) the Chinese Academy of Sciences, 169; (ii) Ocean University of China, 65; (iii) Ministry of Education China, 47; Pilot National Laboratory of Marine Science and technology, 42; and Zheijian University, 38. The United States and Japan were the second- and third-placed countries in terms of publications, respectively. When considering the population of the different countries, South Korea and then Spain and Italy were the most productive countries (population accessed from https://www.worldometers.info/world-population/population-by-country/ accessed on 15 June 2021). 

In addition, in terms of subject areas, most of the publications were included within the group of Agricultural and Biological Sciences and Biochemistry, Genetics, and Molecular Biology. One reason is that many efforts are being undertaken to increase the capacity of microalgae to produce and accumulate astaxanthin by the genetic improvement of strains through genetic engineering [14,15,16,38]. Because of the many health benefits of consuming natural astaxanthin, which will be discussed in the following section, a large number of the publications identified during the last decade were included in the subject areas of Medicine, Immunology, and Microbiology, and, finally, Pharmacology, Toxicology, and Pharmaceutics.

Most of the publications between 2010 and 2020 were published in high-impact journals, as seen in Figure 2D. Most of the studies published during the last decade were published in open access and high-quality journals. The most common journal was the open access journal Marine Drugs, followed by Food Chemistry, Bioresource Technology, Algal Research, and the Journal of Agricultural and Food Chemistry. All of these journals publish research on high-end applications, namely: food, cosmetics, and pharmaceutics. They rank within the top 75% of their respective areas, and four of them have their highest percentile over 90%, which demonstrates the importance and interest related to astaxanthin research.

### 3.2. Industrial Use of Natural Astaxanthin

The bright red color of some fish and crustaceans is one of the major attributes determining quality, purchase intention, and market price. Astaxanthin was first commercialized as an aquafeed ingredient used to increase the astaxanthin content in farmed salmonids and increase the characteristic orange/red color of their flesh [8]. Different microalgae rich in astaxanthin have been used to increase the red color of marine animals. For example, *Monoraphidium* sp. GK12 led to an improvement in the pigmentation of prawns, comparable to what was achieved when using synthetic astaxanthin [39]. Synthetic astaxanthin is more commonly used in aquafeeds when compared to natural astaxanthin or astaxanthin containing microalgae (Table 2). 

However, natural astaxanthin led to improved quality in several reports, and the main advantages and disadvantages of both natural and synthetic astaxanthin have been summarized previously [45]. The main advantages of synthetic astaxanthin include high availability, lower cost, and long lifespan, while the main disadvantages include the use of petrochemical reactants, negative environmental impact, and a complex biosynthesis route that is not sustainable and renewable. In turn, natural astaxanthin has a higher antioxidant capacity, is safer, and has a lower environmental impact. Moreover, natural astaxanthin demonstrated greater pigmentation efficiency than its synthetic counterpart in shrimps fed different diets (25, 50, 75, 100, and 150 mg of astaxanthin per kg) during an 8-week trial [46]. Not only the color of farmed marine animals but also their wellbeing and nutritional quality were improved after the consumption of astaxanthin containing microalgae. Indeed, diets containing microalgae achieved an elevated survival rate when used at low concentrations (5–10 ng of astaxanthin per gram of muscle) over a 4-week period [47]. The carotenoid content of juvenile lobsters fed natural astaxanthin showed an astaxanthin dose-response increase as well as the darkening pigmentation of their exoskeletons [48]. Moreover, a recent study demonstrated that natural astaxanthin promotes the disease resistance of Asian seabass against *Vibrio alginolyticus* infection [49].

Astaxanthin has also been assessed as a feed ingredient for land animals. Dietary supplementation with *Phaffia rhodozyma*, which is a yeast rich in astaxanthin, led to a higher content of total free amino acids and to improved texture and sensorial attributes on broiler chicken meat [50]. The consumption of astaxanthin at 0.25 mg per kg of body weight per day led to increased milk yield in buffaloes and, when combined with prill fat, a better health status was observed, higher than that of prill fat supplementation alone [51]. Several reports demonstrated the potential use of astaxanthin to promote the wellbeing of animals. Recent findings suggested that this compound can affect heat stress and inflammation in broiler chickens and laying hens under high ambient temperatures [52]. Similar results were reported for Sahiwal and Karan Fries heifers, where astaxanthin supplementation alleviated the adverse effect of heat stress and simultaneously improved weight gain and helped in the early attainment of puberty [53].

In terms of human consumption, natural astaxanthin derived from microalgae is generally commercialized as a nutritional supplement as capsules. Several products are currently commercially available, and these include Natural Astaxanthin 5 mg Softgels (Solgar, NJ, USA), Astapure^®^ Astaxanthin Complex (Igennus Healthcare Nutrition, Cambridge, UK), and those listed in Table 1. Food products enriched in astaxanthin have also been formulated, and the results reported to date suggest great potential for further development. For example, raw and cooked lamb patties containing astaxanthin showed reduced lipid oxidation during storage, even improving the performance of metabisuplhite (450 ppm) and ascorbate (500 ppm) [54]. In that same study, not only the quality but also the consumer visual preference was increased after the addition of astaxanthin into the patties [54]. In a different study, the incorporation of 10 mg of astaxanthin per kg of chicken steaks led to minimal lipid oxidation during storage and the preservation of microbiological quality [55]. Similar results were obtained in a different study, where the use of 0.30 and 0.45 g of *H. pluvialis* per kg of minced pork meat delayed lipid oxidation and improved color stability as well as consumer acceptance on the last day of storage [56]. In a different study, astaxanthin encapsulated with zein and oligochitosan was used to increase the antioxidant activity of liquor, apple vinegar, and rice vinegar. The encapsulation of astaxanthin increased the products’ storage stability, especially in terms of stability against ultraviolet light [57]. Because of the health benefits of consuming astaxanthin, this pigment can be used to formulate functional foods that are sold at higher prices and have gained increased consumer interest, especially foods containing marine ingredients [58]. Moreover, the search for and utilization of natural pigments is a top trend in the food industry, especially if the pigment can also act as a radical scavenger [59,60].

Astaxanthin is also being increasingly used in the cosmetics industry. Masks with natural antioxidant are highly appreciated. In a recent study, a mask containing astaxanthin was compared against another mask formulated using vitamin E and demonstrated a superior antioxidant capacity [61]. In that study, the half-life of the mask was calculated as 70 weeks [61]. The same half-life value was reported for an astaxanthin essence stored at 4 °C and produced from Jerusalem artichoke using *Phaffia rhodozyma* [62]. Cosmetics containing microalgae are available in the market, such as the products Bloom Orchid Face Cream or Green Vitamin Concentrate Serum (Freshly Cosmetics, Barcelona, Spain) and Astaxanthin Collagen All-in-One Gel (DHC, Tokyo, Japan), which is a facial moisturizer commercialized in the US as “powdered by astaxanthin, a natural antioxidant proven to be 6000 times more effective than vitamin C” (https://www.dhccare.com/astaxanthin-collagen-all-in-one-gel.html accessed on 15 June 2021). Although the number of cosmetics containing astaxanthin is still limited, because of the extremely high radical scavenging capacity of this molecule, it is likely that further studies will be conducted and products launched into the market.

### 3.3. Health Benefits of Natural Astaxanthin

**Figure 3:**
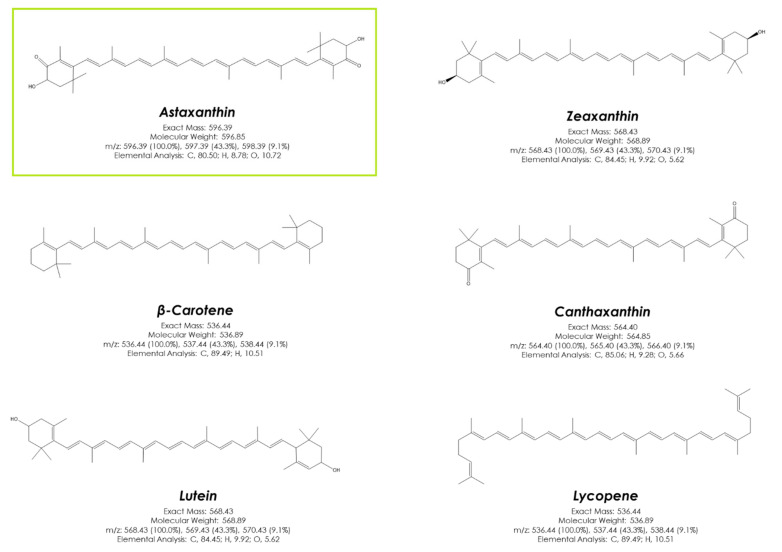
Chemical structure of industry-relevant carotenoids. Astaxanthin, the most potent natural antioxidant is highlighted in green.

Free radicals and reactive oxygen species are naturally produced during normal aerobic metabolism. However, when oxidative molecules are accumulated in excess, they may react with cellular components, causing protein and lipid oxidation or DNA damage, which are associated with various diseases [63]. A correct balance between oxidants and antioxidants, which act as free radical scavengers, is necessary for proper physiological functioning. Antioxidants can be produced by the human body in situ, but most of them, such as carotenoids, are incorporated through diet [64]. Astaxanthin is related to other carotenoids, such as zeaxanthin, lutein, or β-carotene (Figure 3). However, the presence of a keto- and a hydroxyl group on each end of astaxanthin leads to a higher bioactivity of astaxanthin [8]. Indeed, astaxanthin has the highest antioxidant capacity reported for a natural compound, higher than that of other carotenoids [65]. For example, it is 100 times more antioxidant than vitamin E in neutralizing singlet oxygen [66]. 

**Figure 4:**
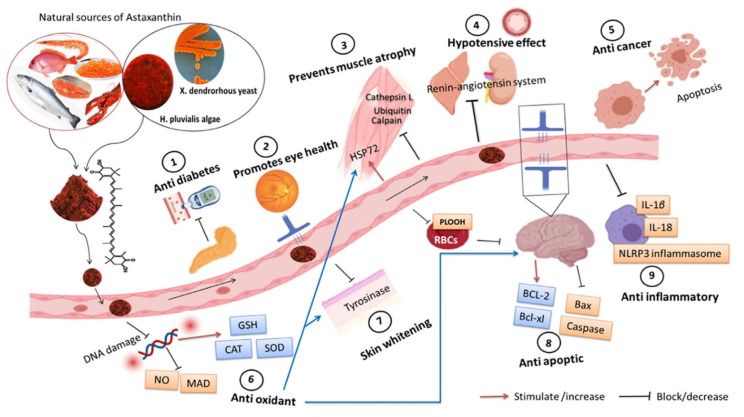
Main effects of astaxanthin. Figure reprinted from [67] with permission from Elsevier.

Most of the health benefits of consuming astaxanthin are attributed to its strong antioxidant capacity. The recent findings are summarized in Table 3, and the main effects of astaxanthin are shown in Figure 4. An important challenge is that, to act as antioxidant in vivo, astaxanthin would have to be transported to the right place and in sufficient quantities to exert a beneficial effect. The bioavailability of bioactive compounds is generally overlooked in scientific literature. Because of the rigid cell wall of microalgae, cell wall disruption steps are generally necessary to liberate intracellular bioactives [60]. Polar carotenoids, such as astaxanthin, tend to have higher bioavailability than apolar compounds, such as lycopene, although the bioavailability of carotenoids is generally low [9]. The antioxidant capacity of astaxanthin was explored in vivo, demonstrating that astaxanthin consumption (2 mg·kg^−1^·day^−1^) exhibited higher antioxidant enzymes, such as catalase or superoxide dismutase, in the brains of mice [67]. Astaxanthin is able to promote the activity of these enzymes, therefore boosting the brain’s glutathione level [67]. Not only bioavailability but the resistance of bioactivity to food processing and storage should also be considered. Only a limited number of studies assessed the effect of storage on the stability of natural astaxanthin. In this regard, a previous report concluded that the optimal processing and storage conditions of *H. pluvialis* biomass were freeze-drying followed by vacuum-packed storage at −20 °C [68]. Further studies on the storage and processing stability of natural astaxanthin are needed.

Astaxanthin can be used to prevent or treat inflammation, a complex sequence of immune responses that take place as defense mechanisms or reactions to bodily injuries [77]. Uncontrolled inflammation reactions induce damage to host cells and tissues and play an important role in several neurodegenerative conditions. Astaxanthin can stop the onset of inflammation and, therefore, play an important role in the prevention of disorders of the central nervous system [78,79]. This valuable carotenoid also decreases gastric inflammation caused by bacterial infections and has an impact on intestinal microbiota, minimizing inflammation and oxidative stress at local and systemic levels in mice [80]. Astaxanthin consumption has been targeted as a potential strategy to mitigate Parkinson’s disease, Alzheimer’s disease, depression, and neuropathic pain, among other disorders [81]. It was reported that microalgae derived astaxanthin can inhibit the expression or production of cytokines and inflammatory mediators and inhibit the expression of inducible nitric oxide synthase and cyclooxygenase-2, with implications for such diseases as brain inflammatory disease, atherosclerosis, or inflammatory bowel disease, among others [9]. Moreover, in a different study, astaxanthin preserved redox-sensitive and key structures of lymphocytes, which could have been caused by its anti-inflammatory activity [82]. It has been suggested that astaxanthin can decrease oxidative stress and inflammation and also enhance the immune response in humans. The consumption of astaxanthin may also affect the immune system as the administration of astaxanthin at a dosage of 60 and 120 mg per kg of body weight prevented intestinal mucosa damage in cyclophosphamide-induced immunodeficient mice [83]. These results must be taken with caution as the doses tested are much higher than the acceptable daily intake of 0.2 mg per kg of body weight set by the EFSA in 2020 [5]. In a different study, an ELISA analysis revealed that astaxanthin could enhance interferon production in response to lipopolysaccharide or concanavalin stimulation, suggesting that astaxanthin can modulate lymphocytic immune responses [84].

Moreover, the antimicrobial potential of astaxanthin has direct health implications as this molecule was capable of reducing the bacterial load and gastric inflammation in mice infected with *Helicobacter pylori* [85,86]. In a different study, the antimicrobial capacity of astaxanthin was validated against different bacteria, including *Escherichia coli*, *Proteus mirabilis*, *Aliivibrio fischeri*, *Enterobacter aerogenes*, and *L. monocytogenes*, suggesting the potential of astaxanthin not only to produce safer foods but also to extend shelf-life [87]. Different antimicrobial polymers based on natural astaxanthin have been developed, with antimicrobial activities demonstrated in vitro with different pathogens minimizing bacterial growth and the formation of biofilms [88]. Extracts of *H. pluvialis* rich in astaxanthin also showed antiviral effects. Indeed, ethanolic extracts of this microalga at concentrations of 75 mg·L-1 inhibited the infection of herpes simplex virus type-1 (HSV-1) on Vero cells (African green monkey kidney cell line) by 85% and also affected HSV-1 intracellular replication [89]. Further studies assessing the antimicrobial and antiviral activity of astaxanthin are necessary as the amount of data currently available in the literature are limited. Other health-promoting properties of astaxanthin reported in the literature, assessed using animal models, include: the preservation of visual function and the protection of retinal ganglion cells apoptosis after ischemic results [90], the promotion of growth and liver function [91], benefits on skin health [92], and anti-apoptotic activity [67].

Overall, astaxanthin demonstrated different bioactivities both in vitro and in vivo in animal models. Further studies are needed to fully elucidate the potential of this valuable bioactive molecule. Assessments of bioavailability and resistance to storage and food processing are of key importance, and data reported to date are scarce. Moreover, in vivo studies with human subjects have been conducted with a limited number of participants, and this needs to be improved in order to achieve health claims for astaxanthin or *H. pluvialis* biomass.

## 4. Astaxanthin Producing Microalgae

Although different microorganisms can produce astaxanthin, natural astaxanthin is industrially obtained from the microalga *H. pluvialis*. Higher plants (with the exception of *Adonis annua*) cannot produce astaxanthin because they lack the enzyme β-carotene ketolasa, which is responsible for introducing keto-moieties to the 4,4′ position of β-ionon rings of carotene and zeaxanthin [93]. Production of *H. pluvialis* mainly occurs in Asia, although there are also production facilities in the US, New Zealand, Chile, Sweden, and Israel, among other countries [41]. This microalga is a freshwater strain that can accumulate astaxanthin up to 3.8–5.0% of dry weight depending on the strain used, the cultivation conditions, and the photobioreactor design [45]. The large-scale production of astaxanthin using *H. pluvialis* is already a reality but still remains a challenge, especially in terms of the high energy requirements and difficult downstream processing. The production of *H. pluvialis* is performed in two steps: a green motile stage and a red non-motile stage. The first stage is equal to that of other microalgal strains, where the microalga is produced in a nutrient-rich medium designed to maximize biomass productivity. At this stage, the most common reactors used are tubular reactors. These are more expensive and complex to operate than open systems. However, because of their capacity to achieve higher biomass concentrations and the high cost of *H. pluvialis* biomass, these systems are the most widely used. Once the maximum concentration is achieved, the biomass is generally (not always) transferred to other reactors where the stress induced by a lack of nutrients leads to the production and accumulation of astaxanthin inside the microalgal cells. Different strategies are being studied to increase the astaxanthin content of *H. pluvialis*, including the addition of exogenous compounds that enhance astaxanthin production [94] or the use of artificial illumination [95]. Artificial illumination is generally prohibited for microalgae production but can be viable for the production of high-end strains and products, such as astaxanthin. One of the strategies with more potential is the genetic modification of *H. pluvialis* strains. Several modified strains have recently been reviewed, achieving, in some cases, an increase in the astaxanthin content higher than 100% [41].

The microalga *C. zofingiensis* can also be used as a feedstock for astaxanthin production and has some advantages when compared to *H. pluvialis*. For example, the growth of *C. zofingiensis* is faster and higher biomass concentrations are able to be achieved. It can be produced phototrophically, heterotrophically, and mixotrophically [93]. The heterotrophic production of *C. zofingiensis* permits reaching higher biomass concentrations. However, the astaxanthin content reached when producing the biomass heterotrophically is generally around 1 mg·g^−1^ [96]. One advantage of *C. zofingiensis* is that this strain can be produced in one step. However, two-step processes have also been developed. In this case, the process consists of an initial fermentation step that allows very high cell densities to be achieved, close to 100 g·L^−1^, and a second step where the astaxanthin content of the cells is increased using outdoor reactors [97]. *Xanthophyllomyces dendrorhous* can accumulate a lower amount of astaxanthin when compared to the above-mentioned strains. Generally, the astaxanthin content of *X. dendrorhous* is below 0.1% of dry weight [93]. However, some *X. dendrorhous* mutants were able to achieve higher concentrations, and the use of phytohormones during biomass production allowed a 24% increase in the astaxanthin content of this microalga to be achieved [98]. Moreover, a recent study concluded that the consumption of *X. dendrorhous* could have the same effect as astaxanthin in preventing obesity caused by a high-fat diet [99]. This strain is being widely studied along with different processes to produce biomass of *X. dendrorhous* using low-cost nutrients, such as fruit and vegetable wastes [100,101], dairy waste [102], and oak leaf extract [103]. In this last study, the supplementation of oak leaf extract with inorganic phosphate (KH_2_PO_4_, 3 mM) resulted in a 1.4-fold increase in the astaxanthin content of the biomass, reaching approximately 2 mg·g^−1^ of dry biomass [103]. Overall, the astaxanthin content of *X. dendrorphous* is lower than that of *H. pluvialis*. However, the possibility of producing the biomass using a low-value nutrient source as well as the potential production of astaxanthin in one step makes the process interesting from a scientific and industrial point of view.

## 5. Conclusions

Natural astaxanthin has several health-promoting properties, most of them as a result of its very high antioxidant capacity. Astaxanthin consumption is thought to prevent and/or control different medical disorders. To date, most of the studies carried out were conducted in vitro and in vivo using animal models. Further in vivo human studies are needed to fully understand the potential of this valuable compound. The results reported so far are promising and suggest a bright future for producers of microalgal astaxanthin. Indeed, natural astaxanthin derived from microalgae is a top trend in food research and in the food industry. Different food products and nutritional supplements are currently available in the market, and the number is expected to increase in the coming years together with the number of publications and processes exploiting astaxanthin. Different microalgae can produce and accumulate astaxanthin, *H. pluvialis* being the most studied and the only one (to the best of the authors’ knowledge) being industrially produced as a source of astaxanthin. The mass production of *H. pluvialis* remains a challenge because of high production costs and the two-step requirement of the process. Other strains, such as *C. zofingiensis* and *X. dendrorhous,* show potential for being used as novel sources for astaxanthin, and several research groups worldwide are currently working on the optimization of their production and downstream processing steps.

## Figures and Tables

**Table 1:** Commercial food products containing natural astaxanthin.

Brand	Company	Description	Astaxanthin Content
Better Foods Astaxanthin	Better Foods GmbH, Göttingen, Germany	Food supplement rich in astaxanthin extracted from *H. pluvialis*. Contains 80 capsules, and the recommended daily intake is two capsules.	4 mg per capsule
Time Health Astaxanthin	Time Health Ltd., Uckfield, UK	Capsules containing 350 mg of powdered *H. pluvialis.* Commercialized as super antioxidant, GMO free, gluten free, and 100% vegan. The recommended daily intake is one capsule.	7 mg per capsule
Natural Astaxanthin	Nutravita Ltd., Maidenhead, UK	Food supplement containing astaxanthin-rich oleoresin from *H. pluvialis*. Softgels commercialized as free from artificial colors and flavors, GMO free, and easy to swallow	0.9 mg per softgel
Astaxanthin 10 mg	Solgar Inc., New Jersey, USA	Softgels containing natural astaxanthin labelled as non-GMO; glute, wheat, and dairy free. Product commercialized as an antioxidant support and a support for a healthy skin	10 mg per softgel
Sila Astaxanthin	Sila, Hsinchu, Taiwan	Food supplement containing astaxanthin extracted from *H. pluvialis* plus vitamins and Omega-3.	12 mg per capsule
Vivanaturals Astaxanthin from microalgae	Viva Naturals Inc., North York, Canada	Softgels sold as GMO free, dairy free, and gluten free that are good for inspiring firm and healthy skin, supporting immune health, and providing antioxidant protection.	4 mg per softgel
Ox Nature Natural Hawaiian Astaxanthin	Ox Nature, Lisbon, Portugal	Softgels commercialized as 100% pure, vegan, GMO free, and wheat free.	4 mg per softgel

**Table 2:** Differences between synthetic and natural astaxanthin.

	Synthetic Astaxanthin	Natural Astaxanthin	Reference
Stereochemistry	(3R,3′R), (3R,3′S), (3S,3′S) optical isomers-1:2:1	(3R,3′R), (3R,3′S), (3S,3′S) optical isomers-1:2:22	[40]
Structure	Non-esterified	More than 95% of the natural astaxanthin molecules are esterified	[40,41]
Industrial uses	Aquaculture feed	Food and dietary supplements, cosmetics, nutraceuticals, aquaculture feed	[41,42]
Market Price	USD 2000 per kg	USD 7000 per kg	[43]
Production	- Wittig reaction of two C15-phosphonium salts with a C10-dialdehyde - Hydroxylation of canthaxanthin. - C10 + C20 + C10 synthesis via dienolether condensation. - Isomerization of a lutein extracted from marigold to zeaxanthin, and then oxidation to astaxanthin.	- - Extraction from algae - - Extraction from yeasts	[44]
Production Price *	800–900 €·kg^−1^	1000–7000 €·kg^−1^	[43]

* Production costs vary significantly between different locations from a theoretical 600–700 €·kg^−1^ in China [13] to 6000–7000 €·kg^−1^ in the Netherlands [11].

**Table 3:** Health benefits of natural astaxanthin when used as food.

Health Benefit	Conditions Studied	Comments	Reference
Protecting against lipid peroxidation	Two 4-mg astaxanthin (Astaxin^®^) capsules daily for 3 months	Astaxanthin consumption decreased the plasma levels of 12- and 15-fatty acids of healthy non-smoking men	[69]
Decreasing TG levels and increasing HDL-cholesterol	12 mg·day^−1^ astaxanthin for 12 weeks	Non-obese subjects with fasting serum triglyceride of 120–200 mg/dL and without diabetes and hypertension, aged 25–60 years.	[70]
Reducing oxidative stress and reversing age-related morphological changes of skin	4 mg·day^−1^ astaxanthin for 4 weeks	The study included 31 volunteers (17 men and 14 women) over the age of 40.	[71]
Improving glucose metabolism and reducing blood pressure	8 mg·day^−1^ astaxanthin for 8 weeks	The study enrolled 44 participants with type 2 diabetes.	[72]
Recovering from mental fatigue	3 mg·day^−1^ and 5 mg·day^−1^ of sesamin for 4 weeks	The study included 24 healthy volunteers.	[73]
Protecting against UV-induced skin deterioration	4 mg·day^−1^ astaxanthin for 10 weeks	The study included 23 healthy participants.	[74]
Maintaining the body’s antioxidant capacity after high-intensity exercise	12 mg·day^−1^ astaxanthin for 4 weeks	The study enrolled 16 male students.	[75]
Decreasing a DNA damage biomarker and enhancing immune response	2–8 mg·day^−1^ astaxanthin for 8 weeks	The study included 42 young healthy females.	[76]

Abbreviations: TG, triglycerides; HDL, high-density lipoprotein; UV, ultraviolet.

## Data Availability

Not applicable.
